# Autophagy Regulates Osteogenic Differentiation of Human Periodontal Ligament Stem Cells Induced by Orthodontic Tension

**DOI:** 10.1155/2022/2983862

**Published:** 2022-10-04

**Authors:** Junyi Zheng, Bowen Xu, Kai Yang

**Affiliations:** Department of Orthodontics, School of Stomatology, Capital Medical University, Beijing, China

## Abstract

Tooth movement is the core of orthodontics. Osteogenesis of the tension side under orthodontic force has great significance on tooth movement and stability, which involves complex mechanical and biological signal transduction. However, the mechanism remains unclear. Through in vitro cell studies, we observed the increased expression levels of osteogenesis-related factors and autophagy-related factors during the osteogenic differentiation of mesenchymal stem cells induced by orthodontic force. The change trend of autophagy-related factors and osteogenesis-related factors is similar, which indicates the involvement of autophagy in osteogenesis. In the study of autophagy-related gene ATG7 silenced cells, the expression level of autophagy was significantly inhibited, and the expression level of osteogenesis-related factors also decreased accordingly. Through drug regulation, we observed that the increase of autophagy level could effectively promote osteogenic differentiation, while the decrease of the autophagy level inhibited this process to some extent. Therefore, autophagy plays an important role in the osteogenic differentiation of mesenchymal stem cells induced by orthodontic force, which provides a novel idea useful for orthodontic treatment in promoting periodontal tissue remodeling and accelerating tooth movement.

## 1. Background

Tooth movement is the core of orthodontics, and the physiological basis is adaptive tissue remodeling of periodontal tissue under mechanical force [1, 2]. As the direct sensor and effector of mechanical force, the cells of periodontal tissues can transform mechanical signals into intracellular and intercellular biochemical signals through a series of cascading reactions, thereby regulating cell morphology, cell proliferation and differentiation, cytokine secretion, and extracellular matrix metabolism and then finally causing the reconstruction of periodontal tissue to achieve the movement of orthodontic teeth [3, 4]. Seo et al. [5] isolated fibroblast-like cells with the ability to form cell clones and multidirectional differentiation and proposed the concept of periodontal ligament stem cells for the first time in 2004. Core binding factor *α*1 (RUNX2) is a key transcription factor regulating bone formation, and it plays a key role in the osteogenic differentiation of mesenchymal stem cells (MSCs). RUNX2 is also a target of mechanical signals during cellular osteogenic differentiation [6, 7]. Alkaline phosphatase (ALP) is a nonspecific phosphate monoesterase that can hydrolyze organic phosphate for apatite crystal deposition, thus providing necessary phosphate acid. Therefore, in research, ALP has been widely considered an early sign of osteoblastic differentiation of MSCs [8]. Osteopontin (OPN) is a secreted glycosylated phosphorylated protein, which belongs to a paracrine factor in osteoblasts. It plays an important role in cell migration, chemotaxis, adhesion, and matrix mineralization, and is a landmark factor in osteoblast differentiation and mid-maturation [9, 10].

Autophagy is a dynamic, continuous, and highly conserved process present in eukaryotes [11, 12]. Depending on the degradation pathway of autophagy substrates, autophagy can be divided into the following three types: macroautophagy (MA), microautophagy, and chaperone-mediated autophagy. Among them, MA, which is characterized by the formation of autophagosomes with a bilayer membrane structure, is the main type of autophagy that has been studied the most. Autophagy can be considered a “circulating factory” of a cell. Moreover, it also plays an important role in stressful states, such as hypoxia, nutritional restriction, and infection [13, 14]. To date, 31 yeast autophagy-related (ATG) genes have been identified, including many homologs of ATG gene products, which have been studied in higher eukaryotes [15]. Atg7 encodes an E1-like enzyme involved in two ubiquitin-like protein-binding systems that are important during autophagosome formation. Microtubule-associated protein light chain 3 (LC3) is a key component of the ubiquitin-like system involved in autophagosome formation. LC3 is expressed as a cytoplasmic protein in most cell types, which is cleaved by Atg4 to produce LC3I, which is subsequently activated by Atg7 in an ATP-dependent manner. The activated LC3I is transferred to Atg3 and finally bound to phosphatidylethanolamine to form LC3II. LC3II is recruits and integrates into the expanding autophagosome and is distributed on the inner and outer surfaces of the autophagosome. Therefore, the increase of LC3 synthesis and processing is a key index to evaluate the level of autophagy in LC3II cells [16, 17].

Recent studies have demonstrated that mechanical stimuli combined with biochemical signals can affect cell fate through modulation of the autophagy pathway. Li et al. [18] established a rat model for orthodontic tooth movement and confirmed for the first time that autophagy is induced by orthodontic load and plays a role in tooth movement. Jiang et al. [19] have reported that mechanical force stimulation increased the expression level of the lateral autophagy protein LC3. Memmert et al. [20] explored the effect of static tensile strain on autophagy level of human periodontal ligament fibroblasts through in vitro experiments; they have reported that high-intensity (20%) and low-intensity (3%) strains can regulate the expression level of autophagy-related mRNA and that autophagy response to static tensile strain is gradual.

Moreover, the level of autophagy changes during the osteogenic differentiation of MSCs and may affect the expression of osteogenesis-related factors. In 2014, Nuschke et al. [21] observed that the undifferentiated MSCs were in a relatively stagnant but highly sensitive state of autophagy. Osteogenic induction using a medium demonstrated a rapidly enhanced autophagy effect, and their analysis suggested that autophagosomes could serve as a source of rapid energy acquisition for cells, which also confirmed the key role of autophagy during differentiation. Nollet et al. [22] have reported that osteoblast mineralization was associated with autophagy induction, while autophagy inhibition resulted in a decreased osteoblast mineralization capacity. Moreover, through transmission electron microscopy and delayed confocal imaging of living cells, autophagy bodies can be used as carriers to secrete apatite crystals into the extracellular matrix. Most current in vitro studies were focused on the observation of the expression level of autophagy in chemically induced osteogenic differentiation and the mechanism of autophagy. However, few studies have reported on the role of autophagy in osteogenic differentiation of human periodontal ligament stem cells (hPDLSCs) induced by strain forces.

Through in vitro cell studies, we observed the expression of autophagy-related factors in the osteogenic differentiation of hPDLSCs induced by mechanical strains. This study also aimed to explore their functional mechanism under distraction and provide new ideas for orthodontics to promote periodontal tissue reconstruction and accelerate tooth movement.

## 2. Materials and Methods

### 2.1. Primary Culture of hPDLSCs

Collected premolars are extracted for orthodontic reasons according to the following criteria: from teenagers aged 10 to 14 years without history of systemic disease, family history of hereditary diseases, smoking history, medication history, and dental, periodontal, and periapical diseases. All participants provided written and informed consent prior to tooth extraction.

The middle section of the periodontal ligament of the root was scraped gently using a sterile surgical blade, digested in a solution of type I collagenase (6 g/L) and type II dispase (8 g/L) in a ratio of 1 : 1 and cultured at 37°C in 5% CO_2_ incubator with alpha-modified Eagle's Medium (*α*-MEM; Gibco, Carlsbad, CA, USA) supplemented with 15% fetal bovine serum (FBS; Gibco, Carlsbad, CA, USA) and 1% penicillin–streptomycin (Gibco, Carlsbad, CA, USA). The colony-derived hPDLSCs at 3 to 5 passages were used for this study.

### 2.2. Characterization

The cells were seeded in a 96-well plate for 1–7 days. Each well was added with 100 *μ*L of medium and 10 *μ*L of CCK8 solution, and the cells were placed in an incubator for 3 h away from light. A growth curve was drawn according to the absorbance values at 450 nm as measured by a microplate reader.

To test the differentiation potential of the hPDLSCs, we performed osteogenic and adipogenic differentiation tests. The cultured cells were seeded in a 6-well plate, and 2 mL of preprepared osteogenic or adipogenic differentiation medium (Cyagen Biosciences, Santa Clara, CA, USA) was added to each well upon reaching 80–100% confluence, following the manufacturer's protocol. After 2–4 weeks of induction, the cells were fixed with 4% formalin, stained with alizarin red or oil red O, and observed using a microscope.

We used flow cytometric analysis to determine the cell phenotype of the hPDLSCs. The cell density was adjusted to 1–5 × 10^6^ cells/mL, and 0.1–10 *μ*g/mL of conjugated primary antibody (Abcam, Cambridge, England) was added. The cells were incubated for 30 min at room temperature in the dark and then analyzed by flow cytometry.

### 2.3. Mechanical Stimulation

Three to five passages of hPDLSCs were seeded on Bioflex culture plates at a cell number of 2 × 10^5^ per well and subjected to a static deformation of 12% [15] for 1–24 h using a Flexercell FX-5000 Strain Unit (Flexcell Corporation, Hillsborough, NC, USA). The corresponding cell culture plates were removed after 1 h, 3 h, 6 h, 12 h, and 24 h of tension application, respectively, and the corresponding cell components were collected in a clean bench and stored at −80°C. The experiment was repeated in triplicate.

### 2.4. Real-Time Polymerase Chain Reaction (PCR)

At the end of each time-course, the media were removed, and the total RNA was extracted from the cells using an RNAso Plus reagent (Takara, Otsu, Shiga Prefecture, Japan), according to the manufacturer's protocol. cDNA synthesis was performed using a PrimeScript™ RT reagent kit (Takara, Otsu, Shiga Prefecture, Japan). The primers used are listed in Table 1. Real-time PCR was performed in a 20 mL reaction system using SYBR Premix Ex Taq™ (Takara, Otsu, Shiga Prefecture, Japan). All genes were normalized to GAPDH, which was performed in triplicate. The experimental results were analyzed using the 2^−△△Ct^ method.

### 2.5. Western Blot Analysis

The total proteins were extracted using an RIPA Lysis Buffer (Solarbio, Beijing, China), and concentration was determined by bicinchoninic acid method. The protein samples were separated by sodium dodecyl-sulfate polyacrylamide gel electrophoresis and transferred to polyvinylidene fluoride membranes that were incubated with a specific primary antibody overnight at 4°C, followed by incubation with a secondary antibody at room temperature for 1 h and visualization using an enhanced chemiluminescence detection system (Millipore, Billerica, MA, USA). The primary antibodies specific to RUNX2, OPN, LC3B, ATG7, and GAPDH were purchased from Abcam (Cambridge, England). Protein expression was quantified using the ImageJ software and normalized to GAPDH.

### 2.6. Assessment of Autophagy Flux

The mRFP–GFP–LC3 adenovirus was transfected according to the manufacturer's instructions. An adenoviral vector (HanHeng, Shanghai, China) was added gently at a multiplicity of infection (MOI) of 30. After 4 h of infection, the medium was added to the complete culture volume. After 6 h of infection, the virus-containing medium was removed and replaced with a fresh medium, and the cells were cultured for 24 h. Subsequently, mechanical force was applied. The autophagosomes and autolysosomes were observed and photographed using a laser confocal microscope, and the images were processed using the ImageJ software. The intensity of the autophagic flux was determined by assessing the number of cells expressing red fluorescent protein (RFP)/green fluorescent protein (GFP).

### 2.7. Transmission Electron Microscope (TEM)

After medium removal and centrifugation, the hPDLSCs were fixed in 4% formaldehyde and 1% glutaraldehyde in 0.1 M phosphate buffer (pH 7.4), immediately. After fixating, dehydrating, embedding, sectioning, and staining, the samples were viewed using a TEM at an acceleration voltage of 80 kV.

### 2.8. RNA Interference

The small hairpin RNA (shRNA) sequence targeting human ATG7 was built by GenScript's shRNA design center (JiMa, Jiangsu, China). A nonsilencing shRNA sequence was used as a control. Recombinant shRNA plasmids were transfected into cells using lentiviruses (JiMa, Jiangsu, China), according to the manufacturer's instructions. A lentivirus was added to the medium, and the medium was replaced with a fresh one after 24 h. After 72 h, the cells were passed and seeded on a BioFlex cell culture plate, and mechanical force was subsequently applied.

### 2.9. Autophagy Modulated by Drugs

Rapamycin (RAPA) is the first known inhibitor of mTOR signaling pathway that inhibits its activity by binding mTORC1, thereby activating downstream autophagy-associated molecular signaling. 3-Methyladenine (3-MA) is a classical inhibitor of autophagy. By inhibiting the formation of Beclin-1–PtdIns 3KC3 complex, the transformation of cytoplasmic soluble form LC3I to autophagosome membrane binding form LC3II is inhibited, thereby inhibiting autophagosome formation. We added RAPA (100 nM) and 3-MA (2 mM) to the cell culture system to regulate autophagy in the cells in the RAPA group and the 3-MA group, respectively. The cells were cultured for 24 h before applying mechanical force.

### 2.10. Statistics

All numerical data are presented as mean ± standard deviation for at least three independent experiments. All statistical analyses were performed using the SPSS statistical software. Data were analyzed using one-way analysis of variance or a paired-sample *t* test. Significance was set at *P* < 0.05.

## 3. Results

### 3.1. Observation and Identification of hPDLSC Morphology

**Figure 1:**
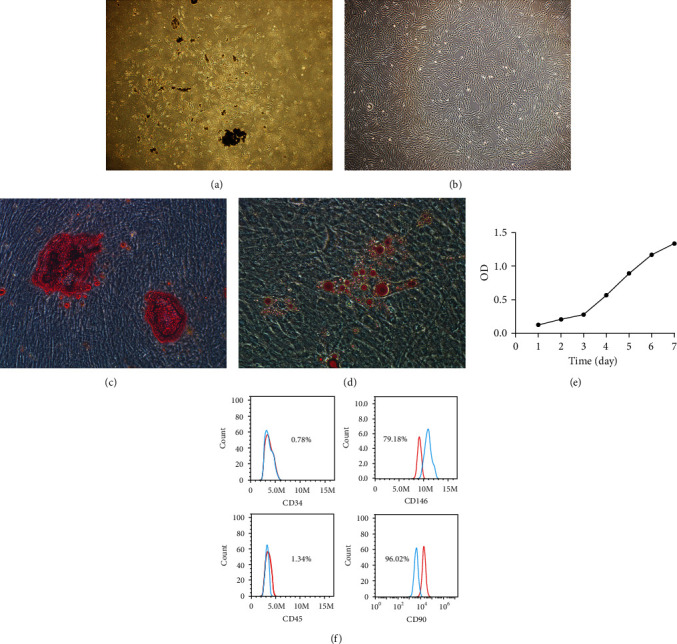
Isolation and characterization of hPDLSCs. (a) Morphology of hPDLSCs after culturing the cells for 7 days. Original magnification ×40. (b) Morphology of the subcultured cells. Original magnification ×40. (c) Cultured hPDLSCs formed alizarin red-positive calcium deposits after 3 weeks of culture in osteogenic induction medium. Original magnification ×100. (d) Cultured hPDLSCs formed oil red O-positive lipid clusters after 3 weeks of culture in adipogenic induction medium. Original magnification ×100. (e) The growth curve is presented in an “S shape.” (f) The positive expression rates of negative markers CD34 and CD45 were 0.78% and 1.24%, respectively; the positive expression rates of positive markers CD146 and CD90 were 79.18% and 96.02%, respectively. OD: optical density; hPDLSCs: human periodontal ligament stem cells.

In total, 19 orthodontic teeth were collected in this experiment. After 4–7 days of primary culture, the cells radially removed from around the tissue blocks, and most of the cells were long spindle-shaped (Figure 1(a)). The subcultured cells grew adherently and had swirling or radial patterns (Figure 1(b)), and the overall growth curve is presented in an “S shape” (Figure 1(e)). After 3 weeks of osteogenic and adipogenic induction, red-stained mineralized nodules and red-stained bead-like lipid droplets were identified under the microscope, confirming that the cultured cells had osteogenic and adipogenic differentiation potential (Figures 1(c) and 1(d)). The surface markers CD34, CD45, CD90, and CD146 of the hPDLSCs were detected by flow cytometry. The positive expression rates of the negative markers CD34 and CD45 were 0.78% and 1.24%, respectively; the positive expression rates of positive markers CD146 and CD90 were 79.18% and 96.02%, respectively (Figure 1(f)).

### 3.2. Cell Viability Detection after Stress Application

**Figure 2:**
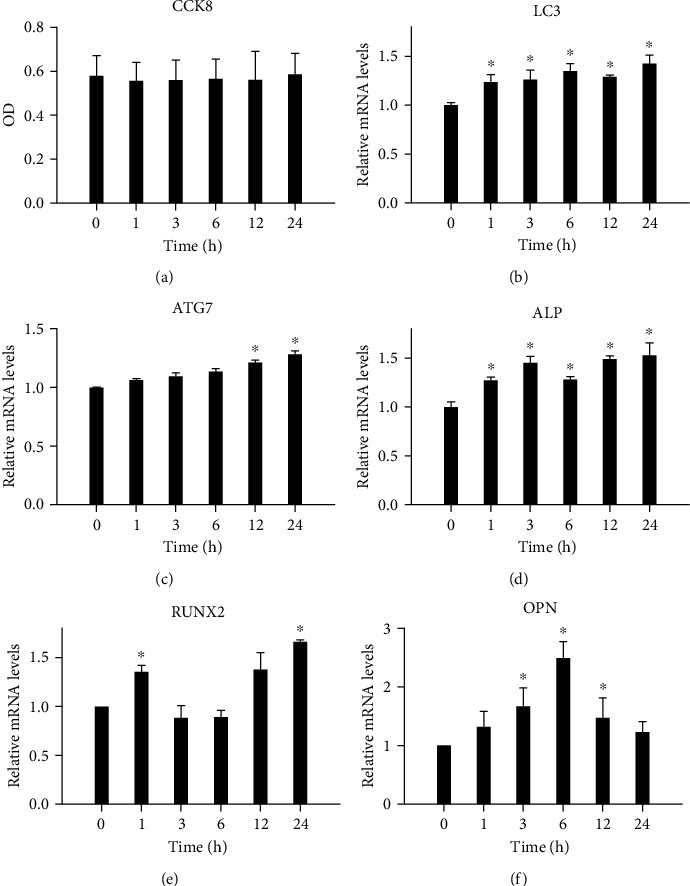
Changes in osteogenic and autophagy levels under mechanical stretch. (a) Cell proliferation activity detected using the CCK-8 kit. (b–f) LC3-, ATG7-, ALP-, RUNX2-, and OPN-relative mRNA expression levels assessed by real-time PCR at 0, 1, 3, 6, 12, and 24 h. ^∗^*P* < 0.05. OD: optical density; PCR: polymerase chain reaction.

Cell proliferation activity was detected using the CCK-8 kit (Figure 2(a)), and no significant difference in cell viability under different durations of stress was observed when 12% static tension was applied to the hPDLSCs.

### 3.3. Changes in the Expression Levels of Autophagy- and Osteogenesis-Related Factors after Tension Application

According to real-time PCR detection, the mRNA expression levels of LC3 (Figure 2(b)) and ATG7 (Figure 2(c)) demonstrated an increasing trend along with the time extension of tension force, when the hPDLSCs were subjected to static tension. The change trend of the mRNA expression levels of ALP (Figure 2(d)) and RUNX2 (Figure 2(e)) along with the time extension of tension force is fundamentally similar to that of autophagy. The difference is that the expression level of OPN (Figure 2(f)) first increased and then gradually returned to the baseline level with the time extension of tension force. ALP at each time point, RUNX2 at 1 and 24 h, and OPN at 3, 6, and 12 h demonstrated significant increases compared with that in the control group (*P* < 0.05).

**Figure 3:**
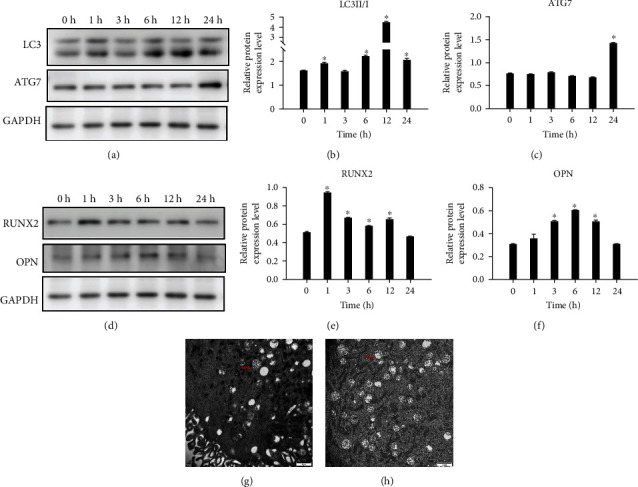
Changes in osteogenic and autophagy levels under mechanical stretch. (a–c) LC3- and ATG7-relative protein expression levels assessed by western blotting at 0, 1, 3, 6, 12, and 24 h. (d–f) RUNX2- and OPN-relative protein expression levels assessed by western blotting at 0, 1, 3, 6, 12, and 24 h. (g, h) Autophagy activation was monitored by transmission electron microscopy, indicated by red arrows. ^∗^*P* < 0.05.

In the western blot analysis, after the hPDLSCs were subjected to static tension, the LC3II/I ratio (Figures 3(a) and 3(b)) increased at 1 h, then gradually increased after 3 h of withdrawal, reached the peak at 12 h, and recovered at 24 h but remained higher than that at baseline. The ATG7 protein expression (Figure 3(c)) did not change significantly with the time extension of tension force, and a significant increase was not observed until 24 h. The ratios of LC3II/I at 1, 6, 12, and 24 h and ATG7 at 24 h were significantly increased than that at baseline (*P* < 0.05). The protein expression levels of OPN (Figures 3(d) and 3(e)) and RUNX2 (Figure 3(f)) demonstrated a trend of first increasing and then gradually returning to the baseline level; expression levels of OPN at 3, 6, and 12 h, and RUNX2 at 1, 3, 6, and 12 h, were significantly higher than those at baseline (*P* < 0.05).

### 3.4. Changes in Autophagic Flux upon Tension Force Application

**Figure 4:**
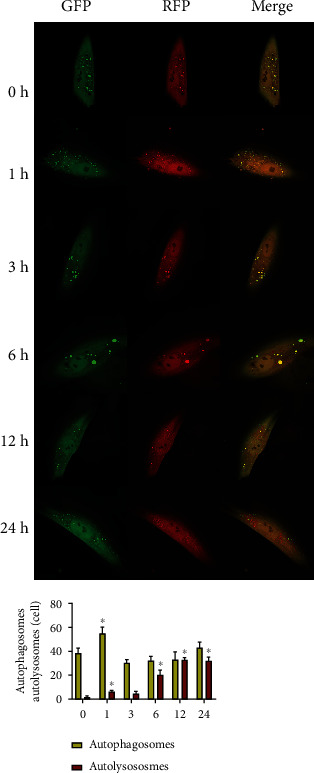
The detection of autophagy flux. ^∗^*P* < 0.05. GFP: green fluorescent protein; RFP: red fluorescent protein.

TEM is currently one of the most reliable and accurate methods to monitor autophagy. As presented in Figure 3(g), autophagic vesicles were observed in the PDLSCs in the control group, and the cells subjected to static tension had more vesicles (Figure 3(h)). In the mRFP–GFP–LC3 adenovirus-transfected cells, RFP and GFP were expressed together with LC3. Prior to autophagosome fusion with lysosomes, they exhibited yellow spots superimposed by red and green spots. When autophagosomes fused with lysosomes to form autophagolysosomes, GFP fluorescence was quenched due to the decrease in pH value, thus indicating the autophagolysosomes as red spots. hPDLSCs were subjected to tension force (Figure 4), and the number of autophagosomes increased significantly after 1 h of tension, which indicated significant difference compared with cells without tension. It decreased after 3 h of tension, and the number of autophagosomes subsequently demonstrated a slow upward trend with prolonged tension force application. Significant differences were observed between the cells without tension force application and those with tension force application at 1 h, 6 h, 12 h, and 24 h.

### 3.5. Lentiviral Transfection Efficiency Detection

**Figure 5:**
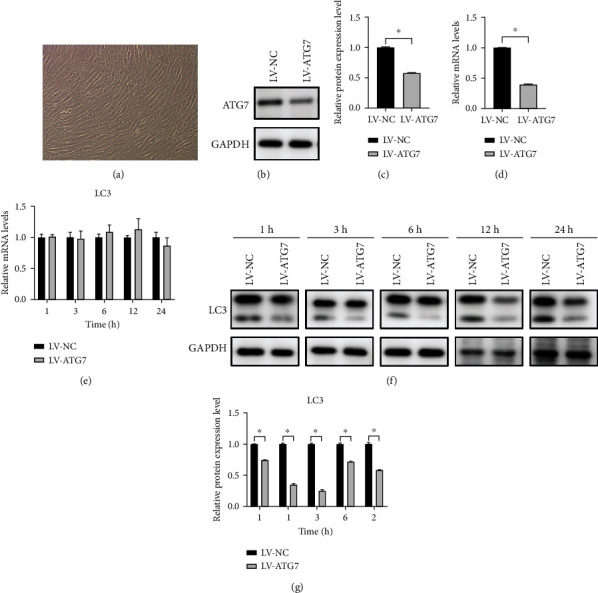
The inhibition of autophagy by knocking down the autophagy-related gene ATG7 of hPDLSCs. (a–d) Lentiviral transfection efficiency detection. (e) LC3-relative mRNA expression levels assessed by real-time PCR in both the LV-NC and LV-ATG7 groups. (f, g) LC3-relative protein expression levels assessed by western blotting in both the LV-NC and LV-ATG7 groups. ^∗^*P* < 0.05. hPDLSCs: human periodontal ligament stem cells.

According to the preexperimental results, the conditions of MOI = 200 and polybrene = 5 *μ*g/mL were used for formal experiments. After transfection of shRNA-ATG7, the morphology of hPDLSCs did not change significantly (Figure 5(a)). The results of the real-time PCR (Figure 5(d)) indicated that in the hPDLSCs transfected with shRNA-ATG7, the mRNA expression level of ATG7 was significantly reduced to approximately 1/3 of the expression in the control cells. The western blot results (Figures 5(b) and 5(c)) revealed that the expression level of ATG7 protein also decreased significantly to approximately 1/2 of the expression in the control cells.

### 3.6. Effects of ATG7 Knockdown on Autophagy

We used the lentiviral shRNA interference vector system to knock down the autophagy-related gene ATG7 in hPDLSCs. A static strain causing 12% cell deformation rate was applied to the stable cell line obtained. Real-time PCR detection (Figure 5(e)) revealed that the mRNA expression level of LC3 was not significantly different from that of the control group at each time point of tension force application. In the western blot analysis (Figures 5(f) and 5(g)), the LC3II/I ratio of the hPDLSCs transfected with shATG7 was significantly lower than that of the control group at each time point (*P* < 0.05).

**Figure 6:**
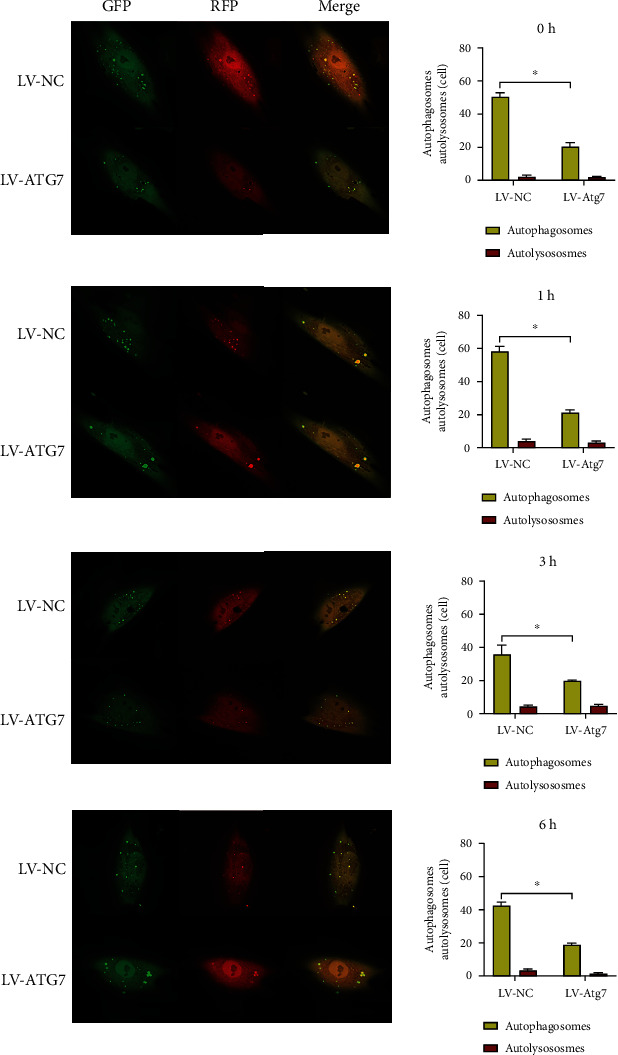
The detection of autophagy flux after knocking down the autophagy-related gene ATG7. GFP: green fluorescent protein; RFP: red fluorescent protein.

**Figure 7:**
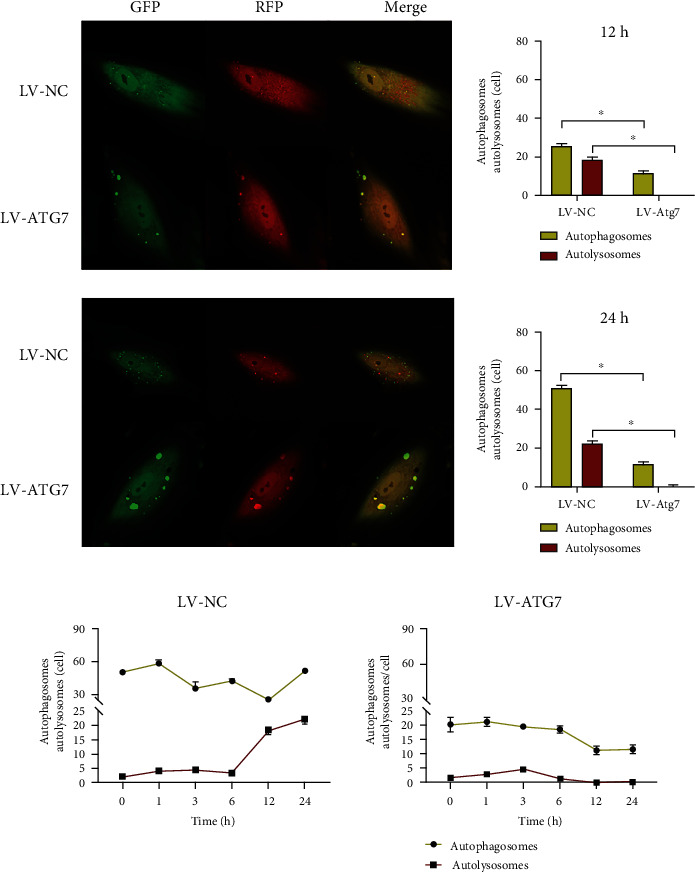
The detection of autophagy flux after knocking down the autophagy-related gene ATG7. GFP: green fluorescent protein; RFP: red fluorescent protein.

After the ATG7 gene was knocked down (Figures 6 and 7), the number of autophagosomes in hPDLSCs was significantly reduced compared with that in the control group cells under different durations of tension force (*P* < 0.05). With the time increase of tension force, the number of autophagosomes did not demonstrate an increasing trend, but a gradually decreasing one. At 12 h and 24 h of tension force, no autophagolysosomes were observed in the cells, and the autophagic flux was significantly inhibited and interrupted.

### 3.7. Effects of ATG7 Knockdown on the Expression Levels of Osteogenesis-Related Factors

**Figure 8:**
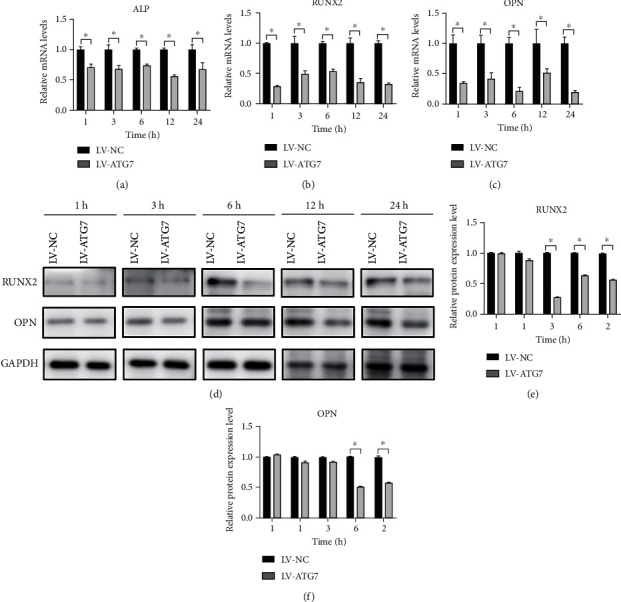
Changes in osteogenic levels after knocking down the autophagy-related gene ATG7. (a–c) ALP-, RUNX2-, and OPN-relative mRNA expression levels assessed by real-time PCR in both the LV-NC and LV-ATG7 groups. (d–f) RUNX2- and OPN-relative protein expression levels assessed by western blotting in both the LV-NC and LV-ATG7 groups. ^∗^*P* < 0.05. PCR: polymerase chain reaction.

The mRNA expressions of ALP (Figure 8(a)), RUNX2 (Figure 8(b)), and OPN (Figure 8(c)) were significantly decreased at each time point compared with the control group (*P* < 0.05). The protein expression level of RUNX2 (Figures 8(d) and 8(e)) was initially lower than that of the control group at 3 h, and the differences were significant at 6, 12, and 24 h (*P* < 0.05). The comparison of OPN expression level (Figure 8(f)) was similar to that of RUNX2. At 3 h onwards, the protein expressions were lower than that of the control group, and the difference was significant at 12 and 24 h (*P* < 0.05).

### 3.8. Effects of RAPA/3-MA on Autophagy

**Figure 9:**
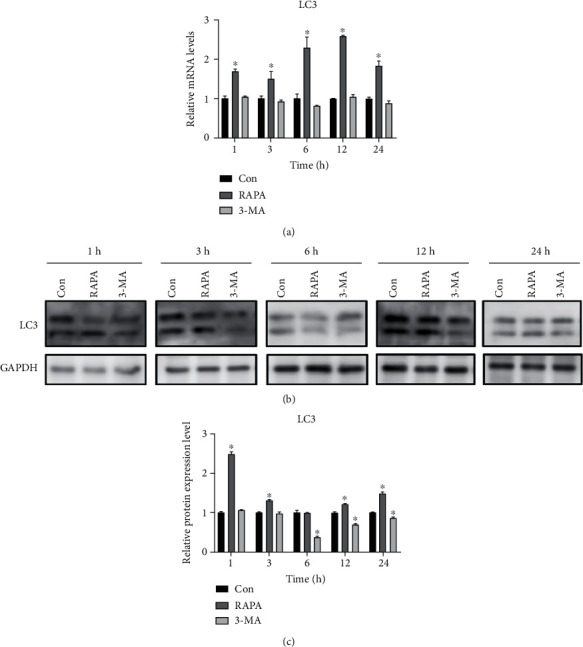
Drug regulation of the autophagy level. (a) LC3-relative mRNA expression levels assessed by real-time PCR in the control, RAPA, and 3-MA groups. (b) LC3-relative protein expression levels assessed by western blotting in the control, RAPA, and 3-MA groups. ^∗^*P* < 0.05. RAPA: rapamycin; 3-MA: 3-methyladenine; Con: control; PCR: polymerase chain reaction.

**Figure 10:**
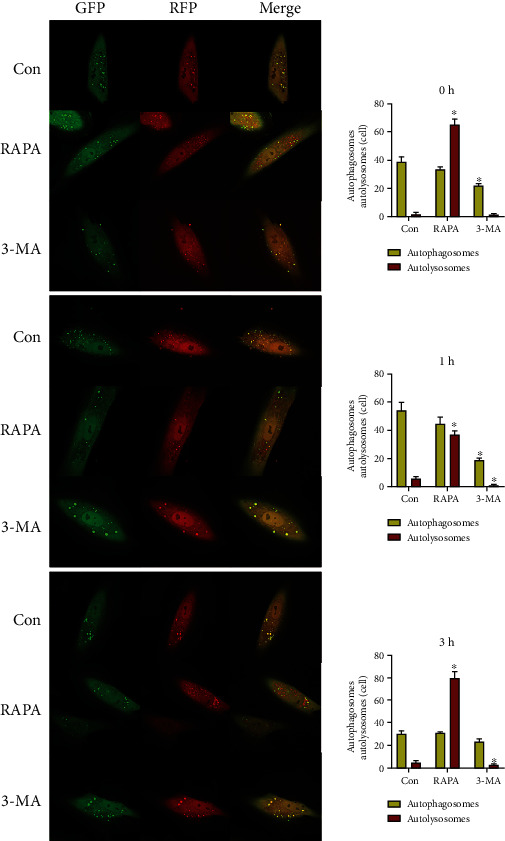
The detection of autophagy flux after drug regulation. ^∗^*P* < 0.05. GFP: green fluorescent protein; RFP: red fluorescent protein; RAPA: rapamycin; 3-MA: 3-methyladenine; Con: control.

**Figure 11:**
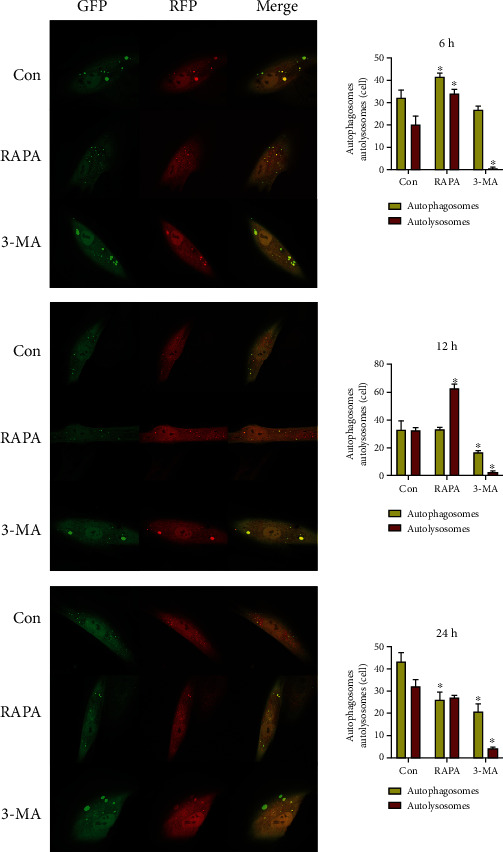
The detection of autophagy flux after drug regulation. ^∗^*P* < 0.05. GFP: green fluorescent protein; RFP: red fluorescent protein; RAPA: rapamycin; 3-MA: 3-methyladenine; Con: control.

We used RAPA, an autophagy promoter, and 3-MA, an autophagy inhibitor, to regulate autophagy level, and applied static traction at 12% cell deformation rate. Real-time PCR detection (Figure 9(a)) indicated that the mRNA expression levels of LC3 in the RAPA group were significantly higher than those in the control group at each time point after loading (*P* < 0.05). The western blot detection revealed (Figures 9(b) and 9(c)) that the LC3II/I ratio of the RAPA group was significantly higher than that of the control group at each time point except at 6 h (*P* < 0.05). The number of autophagy bodies (Figures 10 and 11) significantly increased at 6 h and significantly decreased at 24 h compared with that in the control group (*P* < 0.05). The number of autophagolysosomal lysosomes in the RAPA group with and without different loading durations, except for that at 24 h, was significantly higher than that in the control group (*P* < 0.05).

In the 3-MA group, the LC3 mRNA expression (Figure 9(a)) at each time point was not significantly difference from that of the control group. The western blot detection revealed (Figures 9(b) and 9(c)) that the ratio of the LC3II/I in the 3-MA group was significantly lower than that in control group from 6 h (*P* < 0.05). The number of autophagosomes in the 3-MA group (Figures 10 and 11) with and without different loading durations was significantly decreased compared with that of the control group at 0, 1, 12, and 24 h (*P* < 0.05). The number of autophagolysosomes in the 3-MA group under different loading durations significantly decreased compared with that in the control group (*P* < 0.05).

### 3.9. Effects of RAPA/3-MA on the Expression Levels of Osteogenesis-Related Factors

**Figure 12:**
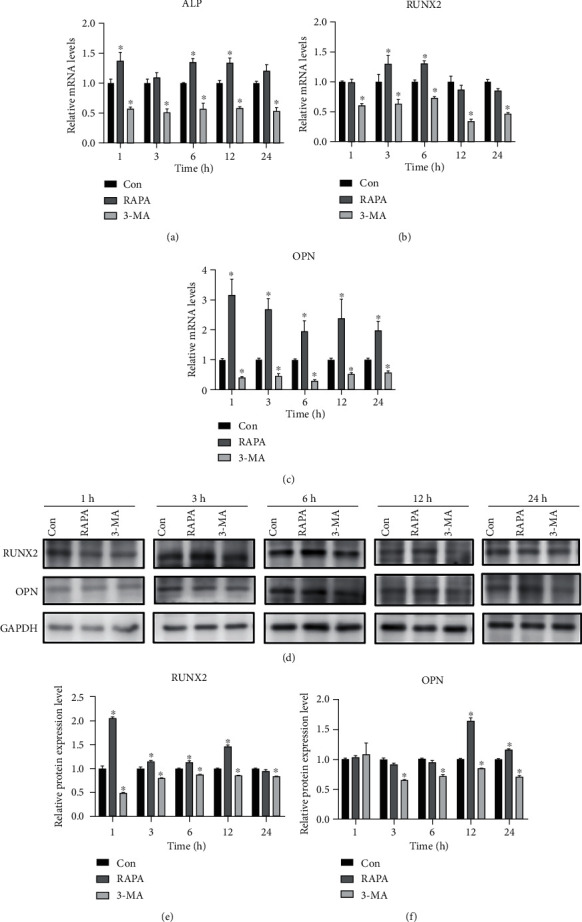
Changes in osteogenic levels after drug regulation. (a–c) ALP-, RUNX2-, and OPN-relative mRNA expression levels assessed by real-time PCR in the control, RAPA, and 3-MA groups. (d–f) RUNX2- and OPN-relative protein expression levels assessed by western blotting in the control, RAPA, and 3-MA groups. ^∗^*P* < 0.05. RAPA: rapamycin; 3-MA: 3-methyladenine; Con: control; PCR: polymerase chain reaction.

Real-time PCR detection demonstrated that the mRNA expression level of ALP (Figure 12(a)) in the RAPA group was higher than that in the control group at each time point, and the difference was significant at 1, 6, and 12 h (*P* < 0.05). The mRNA expression level of RUNX2 (Figure 12(b)) was significantly higher than that of the control group at 3 and 6 h (*P* < 0.05). The mRNA expression levels of OPN (Figure 12(c)) were significantly higher than those in the control group at each time point after loading (*P* < 0.05). The western blot detection revealed that the expression level of RUNX2 (Figures 12(d) and 12(e)) was significantly higher than that in the control group at each time point except at 24 h (*P* < 0.05). OPN expression (Figure 12(f)) was significantly higher than that in the control group at 12 h and 24 h after loading (*P* < 0.05).

In the 3-MA group, the mRNA expression levels of ALP (Figure 12(a)), RUNX2 (Figure 12(b)), and OPN (Figure 12(c)) at each time point were significantly decreased than those in the control group (*P* < 0.05). The western blot detection revealed that RUNX2 (Figures 12(d) and 12(e)) was significantly lower than that in the control group at each time point (*P* < 0.05). Additionally, OPN expression (Figure 12(f)) was significantly lower than that in the control group at each time point except at 1 h (*P* < 0.05).

## 4. Discussion

During orthodontic treatment, adaptive remodeling of periodontal tissue occurs under continuous and appropriate orthodontic loading, followed by directional movement of orthodontic teeth under loading guidance, which involves complex mechanical and biological signal transduction. However, the mechanism remains unclear. In our study, we observed that under mechanical stretch, the expression levels of osteogenesis-related factors and autophagy-related factors were both increased. When autophagy was inhibited through gene silencing technology, the expression level of osteogenesis-related factors also decreased. Through drug regulation, the increase of autophagy level can effectively promote osteogenic differentiation under tension, while the decrease of autophagy level inhibits this process to some extent.

Studies have reported that mechanical traction can promote osteogenic differentiation of MSCs and the expression of osteogenesis-related factors [23–26]. Here, the mRNA and protein expression levels of osteogenesis-related factors were increased after loading, with significant differences at different time point compared with that in the control group. Altogether, it was confirmed that 12% static tension in this experiment could promote osteogenic differentiation of hPDLSCs.

The mRNA expression level of LC3 was significantly increased at each time point compared with that of the control group. The western blot analysis revealed that the ratio of LC3II/I was significantly increased at 1 h after loading, indicating that the conversion of LC3I to LC3II in hPDLSCs was increased shortly after tension stimulation, and the level of autophagy was rapidly activated. Thus, autophagy may be one of the initial responses of cells to mechanical stimulation, which is similar to the results of Porter [27]. With the extension of loading time, the ratio of LC3II/I gradually increased and then decreased again at 24 h, although it remained slightly higher than the basic level. Moreover, the recovery of autophagy level at 24 h may be due to the gradual adaptation of cells to tensile state by altering the cytoskeleton. Tumminia et al. [28] have reported that cells returned to normal morphology after 24 h of static stretching. Additionally, we detected the expression of autophagy-related gene ATG7 in hPDLSCs. The mRNA expression level of ATG7 in hPDLSCs increased significantly after tension, and the protein level did not increase significantly until 24 h. It has been reported that [29] changes in Atg protein levels with the promotion or inhibition of autophagy were generally not apparent.

We investigated the ultrastructure of PDLSCs with or without tension by transmission electron microscopy, and we observed more vesicles in loaded cells. Autophagy is a highly dynamic process; after the formation, autophagosomes can quickly fuse with lysosomes in a relatively short time (5 min) [30]. In order to detect the autophagy flux, we transfected mRFP–GFP–LC3 double-labeled adenovirus into hPDLSCs before cell loading. The results indicated that the number of autophagosomes and autolysosomes increased significantly, demonstrating an upward trend. The rapid growth of autolysosomes was slightly delayed compared with that of autophagosomes. In conclusion, a mechanical stretch can promote the expression of autophagy in hPDLSC.

In this study, the key autophagy gene ATG7 was knocked down to observe whether the expression level of hPDLSCs osteogenesis-related factors would change due to the inhibition of autophagy under the action of tension. No significant difference in the mRNA expression level of LC3 was observed between the control group and ATG7 group at each time point. Our analysis suggested that this was because ATG7 played a role in the transformation of the LC3 protein from type I to type II. Therefore, the knockdown of ATG7 did not significantly affect the expression of LC3 gene. Kyung [31] has also reported that the expression level of LC3II in Hep3B cells with downregulated ATG7 expression did not demonstrate an upward trend under starvation stimulation. The western blot results revealed that after ATG7 gene knockdown, the LC3II/I ratios were significantly lower than those in the control group, indicating that the formation of autophagy bodies was inhibited to some extent.

Regarding autophagy flow, after ATG7 gene was knocked down, the number of autophagosomes and autophagolysosomal lysosomes was significantly reduced compared with the control group cells under different loading durations, which was similar to the results of Shadab et al. [32]. In the detection of autophagy flow, after transfection of the lentivirus, the number of autophagosomes did not increase due to cell loading, but demonstrated a gradual downward trend. Autophagic lysosomes could still be detected in the cells within 6 h of loading, and the number increased slightly. However, autophagy lysosomes could not be detected in the cells within 12 h to 24 h of loading. We speculated that ATG7 knockdown blocked the formation of autophagosomes to a certain extent, but had no significant inhibitory effect on the fusion of formed autophagosomes and lysosomes. Therefore, in the early stage of loading, the formation of autophagosomes was not affected temporarily, and the number of autophagosomes decreased with the extension of loading time. In the late stage of loading, autophagosomes stored in cells have primarily completed the process of autophagy degradation, so the synthesis of autolysosomes was interrupted, and the autophagy flow was significantly inhibited.

Regarding osteogenic differentiation levels, the mRNA and protein expression levels of ALP, RUNX2, and OPN were significantly lower than those in the control group, which indicated that after ATG7 gene knockdown, the osteogenic differentiation of hPDLSCs under the action of traction was weakened. Yi et al. [33] have also reported that the downregulation of ATG7 expression in osteogenic precursor cells significantly reduced the role of triiodothyronine (T3) in promoting the expression of cell osteogenic markers.

Finally, we added RAPA and 3-MA into the cell culture system to observe whether the drug-regulated autophagy of cells could affect the osteogenic differentiation level of hPDLSCs induced by tension. The expression levels of the LC3 and LC3II/I ratios were increased at most time points after RAPA treatment, indicating that the autophagy level was increased. Regarding autophagy flux, after RAPA treatment, the number of autolysosomes increased significantly compared with that in the control group at all time points except at 24 h. By contrast, the increase in the number of autophagosomes was not very significant. We speculated that RAPA treatment may significantly accelerate the degradation rate of autophagy bodies. Therefore, the increase in the synthesis of autophagy bodies may not be easily detected under the microscope. After 3-MA treatment, the LC3II/I ratio was significantly lower than that of the control group. Additionally, the number of autophagosomes and autolysosomes decreased compared with that in the control group under most loading times. Therefore, RAPA treatment can promote autophagy, while 3-MA treatment can inhibit autophagy.

Regulating autophagy has been confirmed to regulate the osteogenic differentiation level to a certain extent [34–38]. In this experiment, the expression levels of osteogenesis-related factors ALP, RUNX2, and OPN at mRNA and protein levels of hPDLSCs treated with RAPA were significantly increased compared with those of the control group at different loading times. The 3-MA treatment decreased the expression levels significantly. Similar results have also been reported in a study of bone marrow MSC stretch by Zhou et al. [35].

In conclusion, in this study, hPDLSCs were primarily cultured in vitro, and 12% static tension was applied using a Flexcell cell dynamometer to simulate the tension-side cells being pulled during orthodontic tooth movement in vivo. Our results indicated that autophagy played a key regulatory role in osteogenic differentiation induced by mechanical force.

## Figures and Tables

**Table 1:** Primer sequences.

Name	Gene name/NCBI gene ID	Full-length name	Length of the product	Sequence	
ALP	*ALPL* ID: 249	Alkaline phosphatase	104	Forward	ACGAGCTGAACAGGAACAACGT
				Reverse	CACCAGCAAGAAGAAGCCTTTG

RUNX2	*RUNX2* ID: 860	RUNX family transcription factor 2	150	Forward	AGGCAGTTCCCAAGCATTTCATCC
				Reverse	TGGCAGGTAGGTGTGGTAGTGAG

OPN	*SPP1* ID: 6696	Secreted phosphoprotein 1	300	Forward	TTGCTTTTGCCTCCTAGGCA
				Reverse	GTGAAAACTTCGGTTGCTGG

LC3	*MAP1LC3A* ID: 84557	Microtubule-associated protein 1 light chain 3 alpha	140	Forward	TCAGGTTCACAAAACCCGCC
				Reverse	GCGTTTGTGCCAACTGTGAT

ATG7	*ATG7* ID: 10533	Autophagy-related 7	138	Forward	AGCGGCGGCAAGAAATAA
				Reverse	CCAGCCGATACTCGTTCA

GAPDH	*GAPDH* ID: 2597	Glyceraldehyde-3-phosphate dehydrogenase	141	Forward	GGTCACCAGGGCTGCTTTT
				Reverse	GGATCTCGCTCCTGGAAGA

## Data Availability

The data used to support the findings of this study are included within the article.

## References

[1] Masella R. S., Meister M. (2006). Current concepts in the biology of orthodontic tooth movement. *American Journal of Orthodontics and Dentofacial Orthopedics*.

[2] Henneman S., Von den Hoff J. W., Maltha J. C. (2008). Mechanobiology of tooth movement. *European Journal of Orthodontics*.

[3] Ziros P. G., Gil A. P. R., Georgakopoulos T. (2002). The bone-specific transcriptional regulator Cbfa1 is a target of mechanical signals in osteoblastic cells. *Journal of Biological Chemistry*.

[4] Wei F., Wang C., Zhou G. (2008). The effect of centrifugal force on the mRNA and protein levels of ATF4 in cultured human periodontal ligament fibroblasts. *Archives of Oral Biology*.

[5] Seo B. M., Miura M., Gronthos S. (2004). Investigation of multipotent postnatal stem cells from human periodontal ligament. *Lancet*.

[6] Camilleri S., McDonald F. (2006). Runx2 and dental development. *European Journal of Oral Sciences*.

[7] Kawarizadeh A., Bourauel C., Gotz W., Jager A. (2005). Early responses of periodontal ligament cells to mechanical stimulus in vivo. *Journal of Dental Research*.

[8] Wuthier R. E., Register T. C. (1985). *Role of Alkaline Phosphatase, a Polyfunctional Enzyme, in Mineralising Tissues*.

[9] Mckee M., Nanci A. (1996). Osteopontin at mineralized tissue interfaces in bone, teeth, and osseointegrated implants: ultrastructural distribution and implications for mineralized tissue formation, turnover, and repair. *Microscopy Research and Technique*.

[10] Teruyoshi K., Kenichi M., Toru S. (2010). Effect of stretching force on the cells of epithelial rests of Malassez in vitro. *International Journal of Dentistry*.

[11] Ashford T. P., Porter K. R. (1962). Cytoplasmic components in hepatic cell lysosomes. *The Journal of Cell Biology*.

[12] Klionsky D. J. (2008). Autophagy revisited: a conversation with Christian de Duve. *Autophagy*.

[13] Yang Z., Klionsky D. J. (2010). Eaten alive: a history of macroautophagy. *Nature Cell Biology*.

[14] Shen G., Ren H., Shang Q. (2018). Autophagy as a target for glucocorticoid-induced osteoporosis therapy. *Cellular and Molecular Life Sciences*.

[15] Huang J., Klionsky D. J. (2007). Autophagy and human disease. *Cell Cycle*.

[16] Hanada T., Noda N. N., Satomi Y. (2007). The Atg12-Atg5 conjugate has a novel E3-like activity for protein lipidation in autophagy. *Journal of Biological Chemistry*.

[17] Romanov J., Walczak M., Ibiricu I. (2012). Mechanism and functions of membrane binding by the Atg5–Atg12/Atg16 complex during autophagosome formation. *EMBO Journal*.

[18] Li Y., Jacox L. A., Coats S. (2021). Roles of autophagy in orthodontic tooth movement. *American Journal of Orthodontics and Dentofacial Orthopedics*.

[19] Jiang N., He D., Ma Y. (2021). Force-induced autophagy in periodontal ligament stem cells modulates M1 macrophage polarization via AKT signaling. *Frontiers in Cell and Development Biology*.

[20] Memmert S., Damanaki A., Weykopf B. (2019). Autophagy in periodontal ligament fibroblasts under biomechanical loading. *Cell and Tissue Research*.

[21] Nuschke A., Rodrigues M., Stolz D. B., Chu C. T., Griffith L., Wells A. (2014). Human mesenchymal stem cells/multipotent stromal cells consume accumulated autophagosomes early in differentiation. *Stem Cell Research & Therapy*.

[22] Nollet M., Santucci-Darmanin S., Breuil V. (2014). Autophagy in osteoblasts is involved in mineralization and bone homeostasis. *Autophagy*.

[23] Wescott D. C., Pinkerton M. N., Gaffey B. J., Beggs K. T., Milne T. J., Meikle M. C. (2007). Osteogenic gene expression by human periodontal ligament cells under cyclic tension. *Journal of Dental Research*.

[24] Liu M., Dai J., Lin Y. (2012). Effect of the cyclic stretch on the expression of osteogenesis genes in human periodontal ligament cells. *Gene*.

[25] Shen T., Qiu L., Chang H. (2014). Cyclic tension promotes osteogenic differentiation in human periodontal ligaments stem cells. *The International Journal of Clinical and Experimental Pathology*.

[26] Chiba M., Mitani H. (2004). Cytoskeletal changes and the system of regulation of alkaline phosphatase activity in human periodontal ligament cells induced by mechanical stress. *Cell Biochemistry and Function*.

[27] Porter K. M. (2014). MTOR-independent induction of autophagy in trabecular meshwork cells subjected to biaxial stretch. *Cell Research*.

[28] Tumminia S. J., Mitton K. P., Arora J., Zelenka P., Epstein D. L., Russell P. (1998). Mechanical stretch alters the actin cytoskeletal network and signal transduction in human trabecular meshwork cells. *Investigative Ophthalmology & Visual Science*.

[29] Klionsky D. J., Abeliovich H., Agostinis P. (2008). Guidelines for the use and interpretation of assays for monitoring autophagy in higher eukaryotes. *Autophagy*.

[30] Lawrence B. P., Brown W. J. (1992). Autophagic vacuoles rapidly fuse with pre-existing lysosomes in cultured hepatocytes. *Journal of Cell Science*.

[31] Cho K. J., Shin S. Y., Moon H., Kim B. K., Ro S. (2021). Knockdown of Atg7 suppresses tumorigenesis in a murine model of liver cancer-Science Direct. *Translational Oncology*.

[32] Shadab M., Millar M. W., Slavin S. A., Leonard A., Fazal F., Rahman A. (2020). Autophagy protein ATG7 is a critical regulator of endothelial cell inflammation and permeability. *Scientific Reports*.

[33] Yi L., Zhong T., Huang Y., Huang S. (2020). Triiodothyronine promotes the osteoblast formation by activating autophagy. *Biophysical Chemistry*.

[34] Qi M., Zhang L., Yang M. (2017). Autophagy maintains the function of bone marrow mesenchymal stem cells to prevent estrogen deficiency-induced osteoporosis. *Theranostics*.

[35] Zhou Z., Shi G., Zheng X., Jiang S., Jiang L. (2018). Autophagy activation facilitates mechanical stimulation-promoted osteoblast differentiation and ameliorates hindlimb unloading-induced bone loss. *Biochemical and Biophysical Research Communications*.

[36] Luo D., Ren H., Li T., Lian K., Lin D. (2016). Rapamycin reduces severity of senile osteoporosis by activating osteocyte autophagy. *Osteoporosis International*.

[37] Liu F., Fang F., Yuan H. (2013). Suppression of autophagy by FIP200 deletion leads to osteopenia in mice through the inhibition of osteoblast terminal differentiation. *Journal of Bone and Mineral Research*.

[38] Nakamura T., Yamashita M., Ikegami K. (2021). Autophagy facilitates type I collagen synthesis in periodontal ligament cells. *Scientific Reports*.

